# Supplementary material for: Embryonic loss of human females with partial trisomy 19 identifies region critical for the single active X
Source: PLoS One. 2017 Apr 12;12(4):e0170403. doi: 10.1371/journal.pone.0170403 (PMC5389809; doi:10.1371/journal.pone.0170403)

Fig A (related to Fig 2). Posterior gain rate (M:F) plotted serially on chromosome 2 using available genes from DECIPHER. Dashed line depicts equal gain rate.

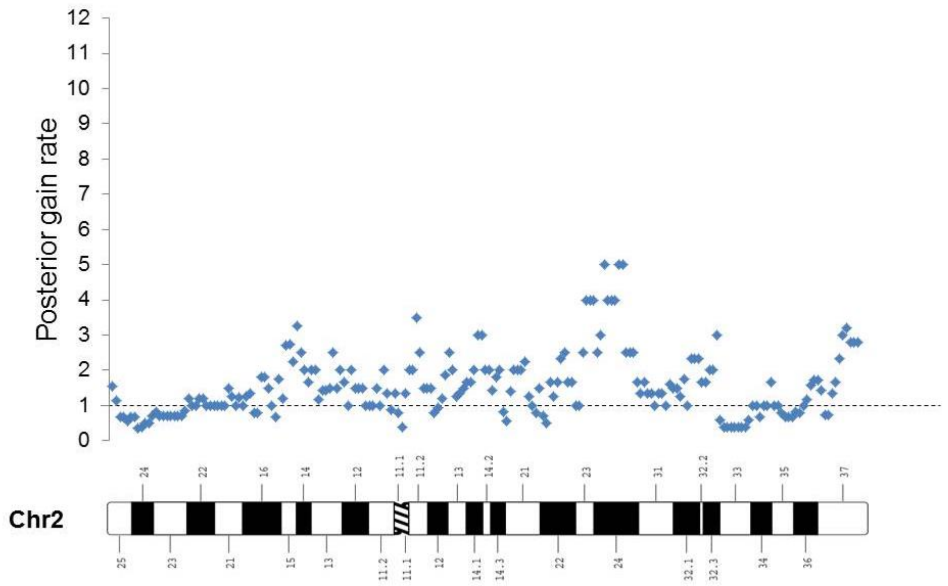

Fig B (related to Fig 2). Posterior gain rate (M:F) plotted serially on chromosome 3 using available genes from DECIPHER. Dashed line depicts equal gain rate.

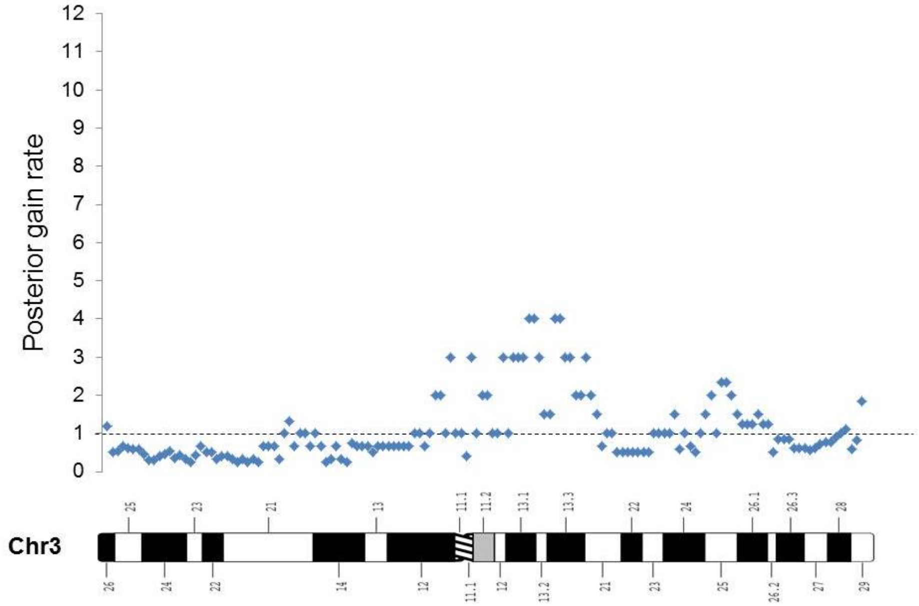

Fig C (related to Fig 2). Posterior gain rate (M:F) plotted serially on chromosome 4 using available genes from DECIPHER. Dashed line depicts equal gain rate.

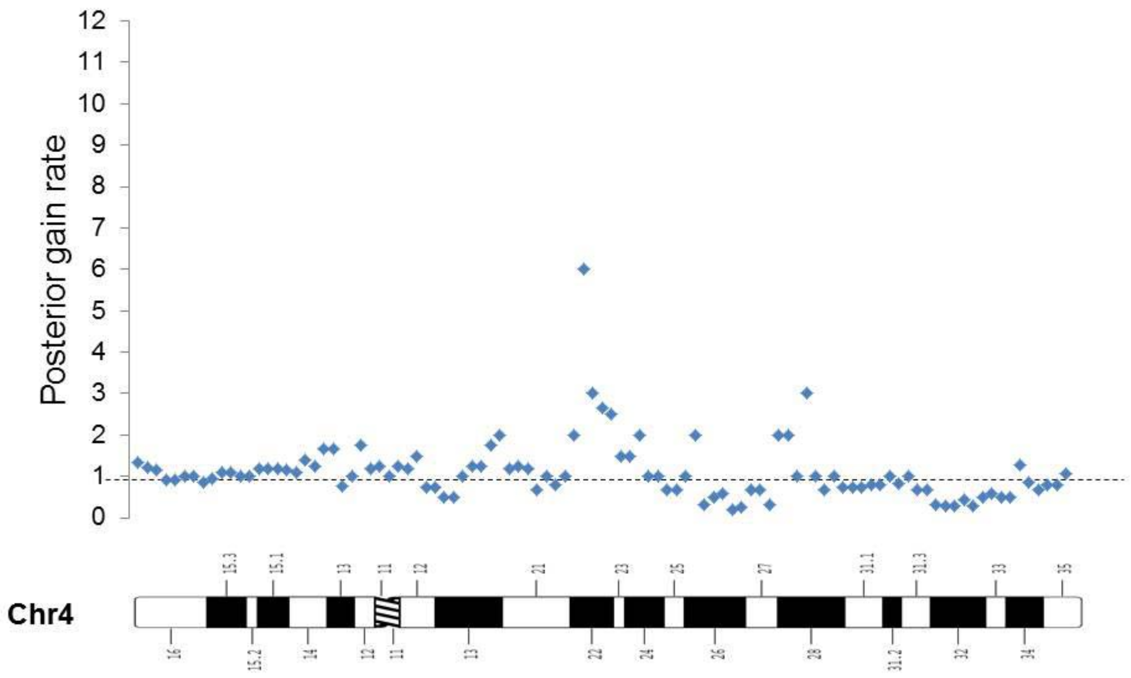

Fig D (related to Fig 2). Posterior gain rate (M:F) plotted serially on chromosome 5 using available genes from DECIPHER. Dashed line depicts equal gain rate.

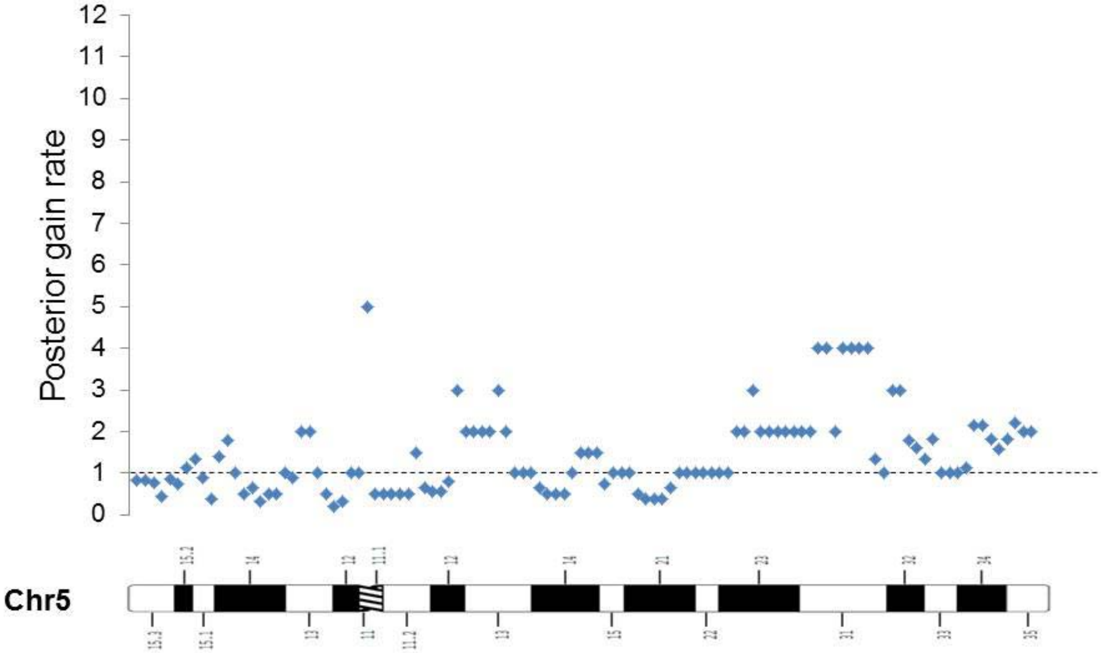

Fig E (related to Fig 2). Posterior gain rate (M:F) plotted serially on chromosome 6 using available genes from DECIPHER. Dashed line depicts equal gain rate.

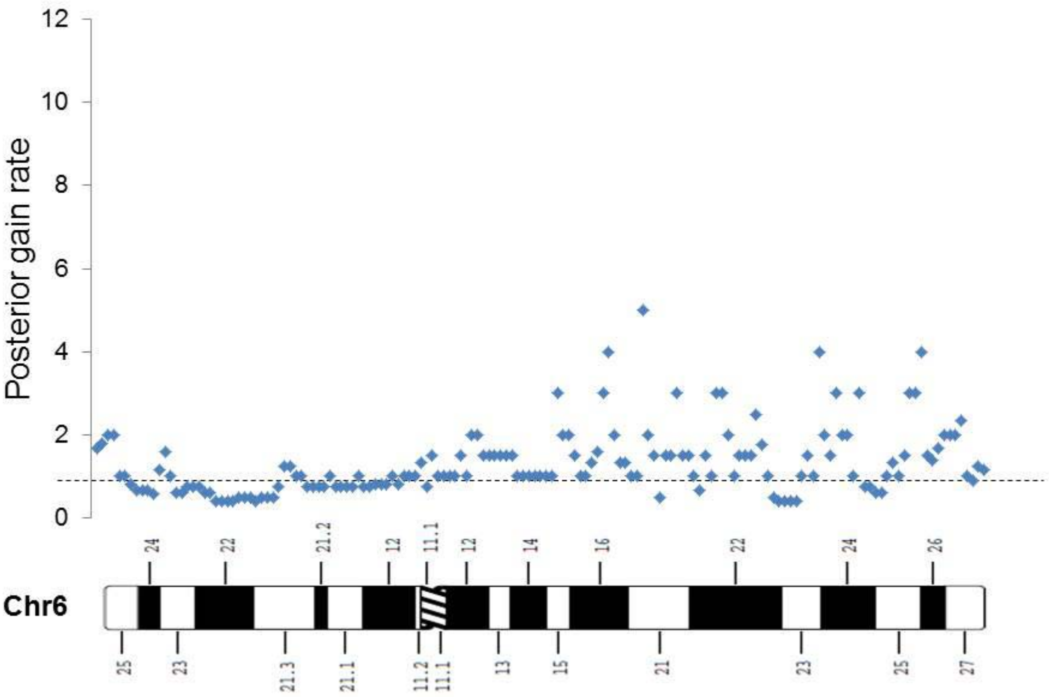

Fig F (related to Fig 2). Posterior gain rate (M:F) plotted serially on chromosome 7 using available genes from DECIPHER. Dashed line depicts equal gain rate.

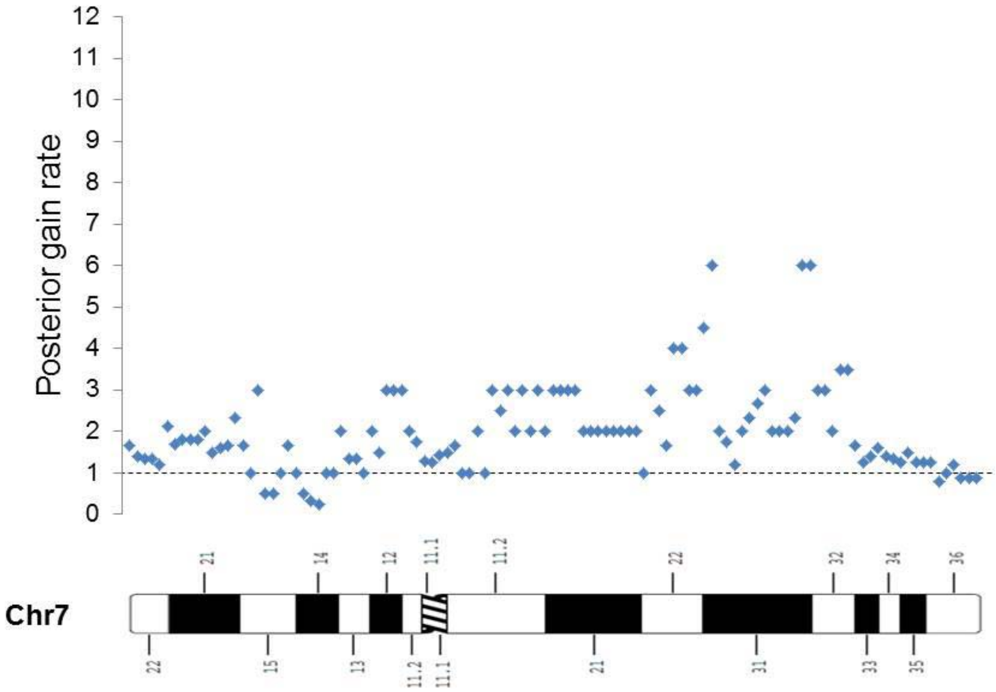

Fig G (related to Fig 2). Posterior gain rate (M:F) plotted serially on chromosome 8 using available genes from DECIPHER. Dashed line depicts equal gain rate.

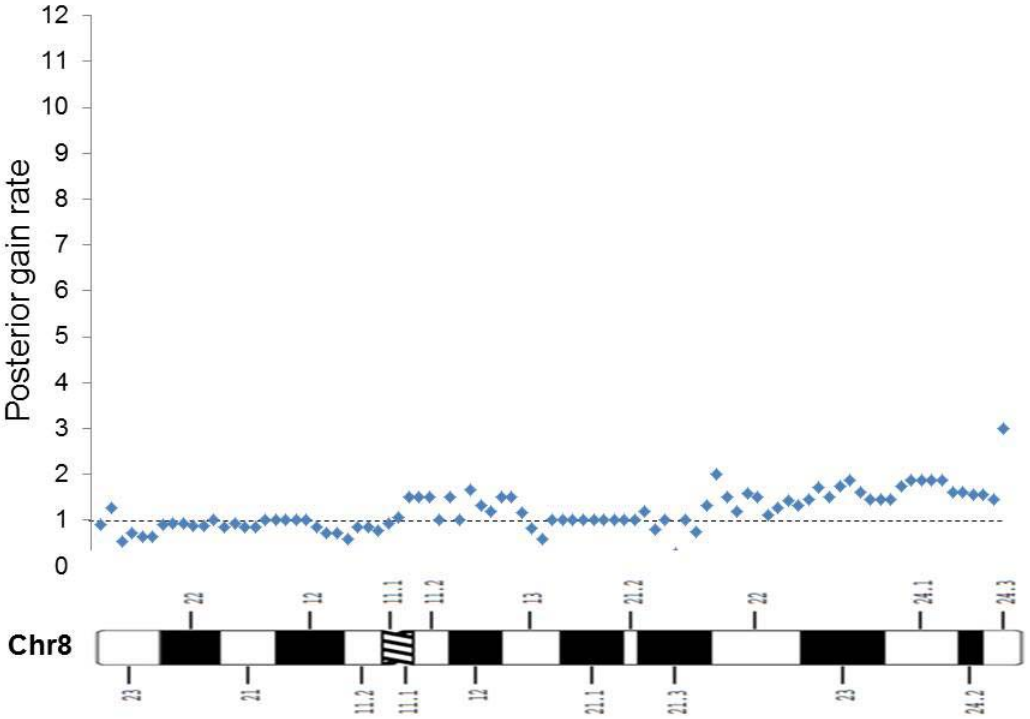

Fig H (related to Fig 2). Posterior gain rate (M:F) plotted serially on chromosome 9 using available genes from DECIPHER. Dashed line depicts equal gain rate.

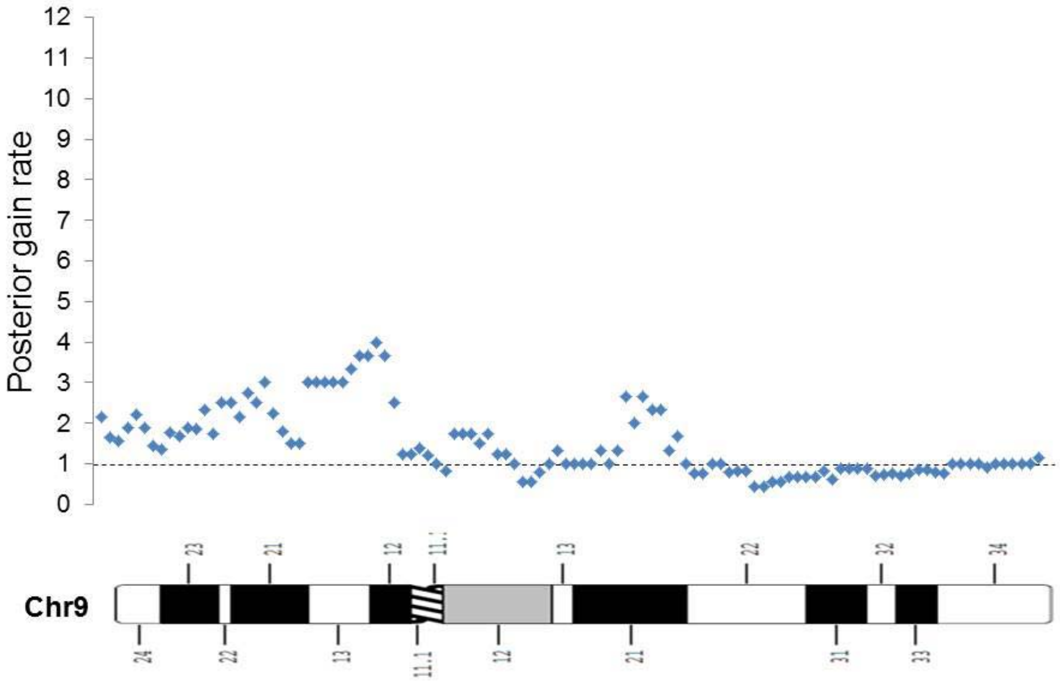

Fig I (related to Fig 2). Posterior gain rate (M:F) plotted serially on chromosome 10 using available genes from DECIPHER. Dashed line depicts equal gain rate.

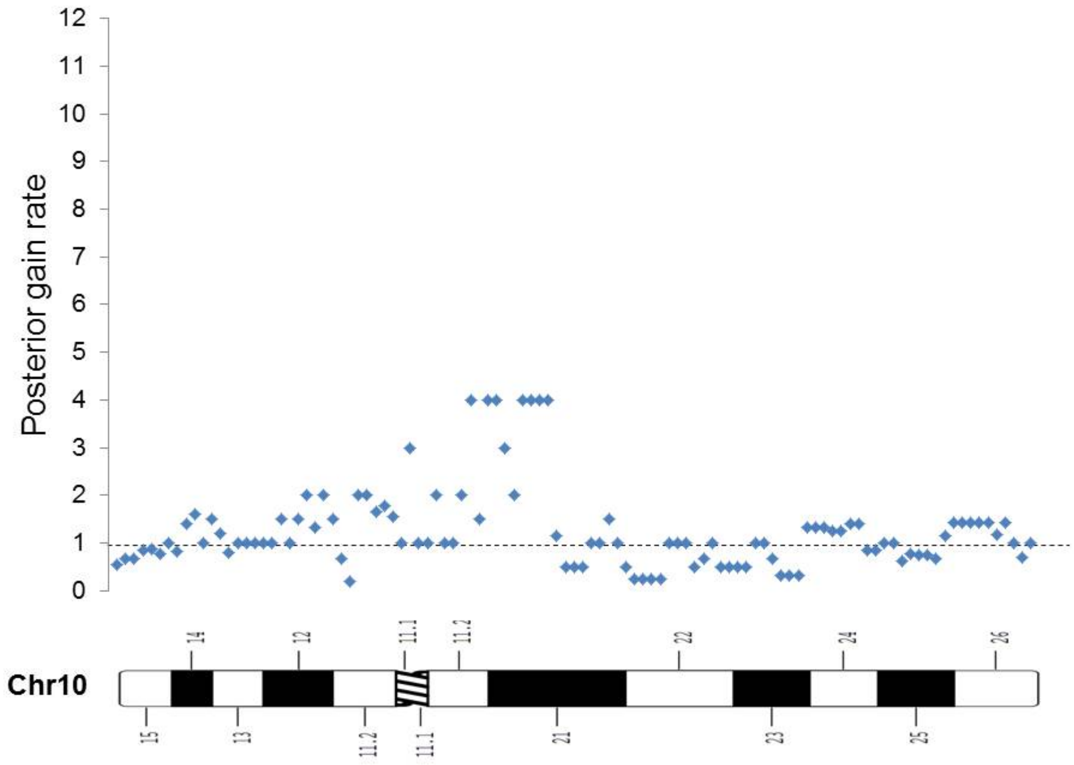

Fig J (related to Fig 2). Posterior gain rate (M:F) plotted serially on chromosome 11 using available genes from DECIPHER. Dashed line depicts equal gain rate.

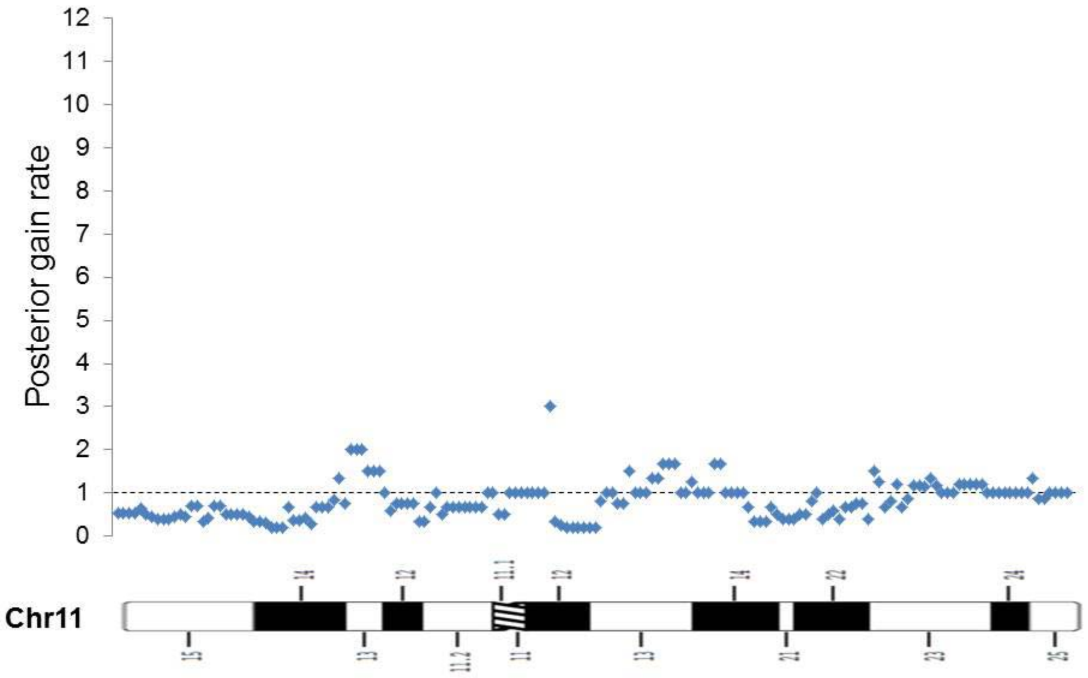

Fig K (related to Fig 2). Posterior gain rate (M:F) plotted serially on chromosome 12 using available genes from DECIPHER. Dashed line depicts equal gain rate.

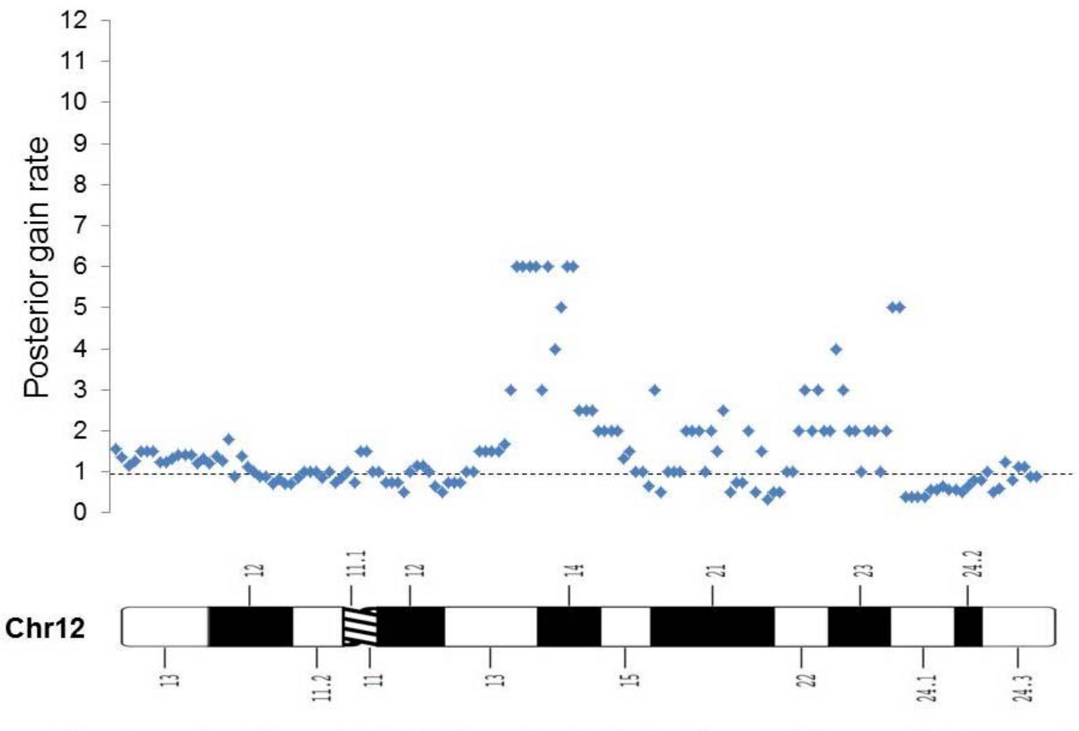

Fig L (related to Fig 2). Posterior gain rate (M:F) plotted serially on chromosome 13 using available genes from DECIPHER. Dashed line depicts equal gain rate.

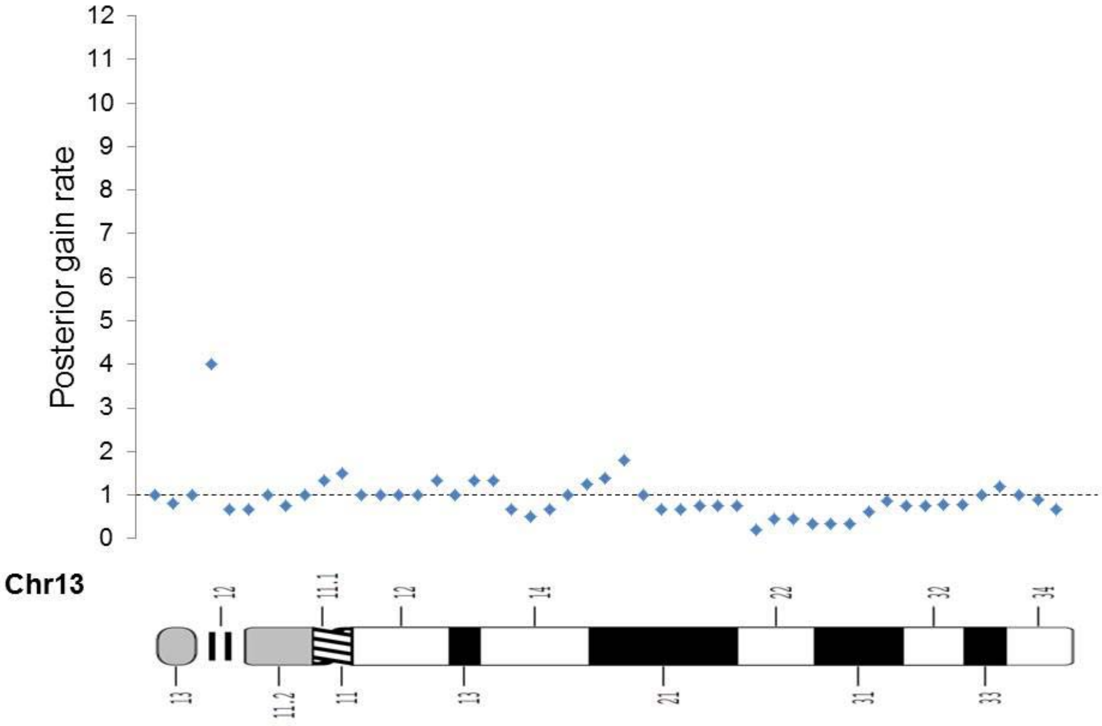

Fig. M (related to Fig 2). Posterior gain rate (M:F) plotted serially on chromosome 14 using available genes from DECIPHER. Dashed line depicts equal gain rate.

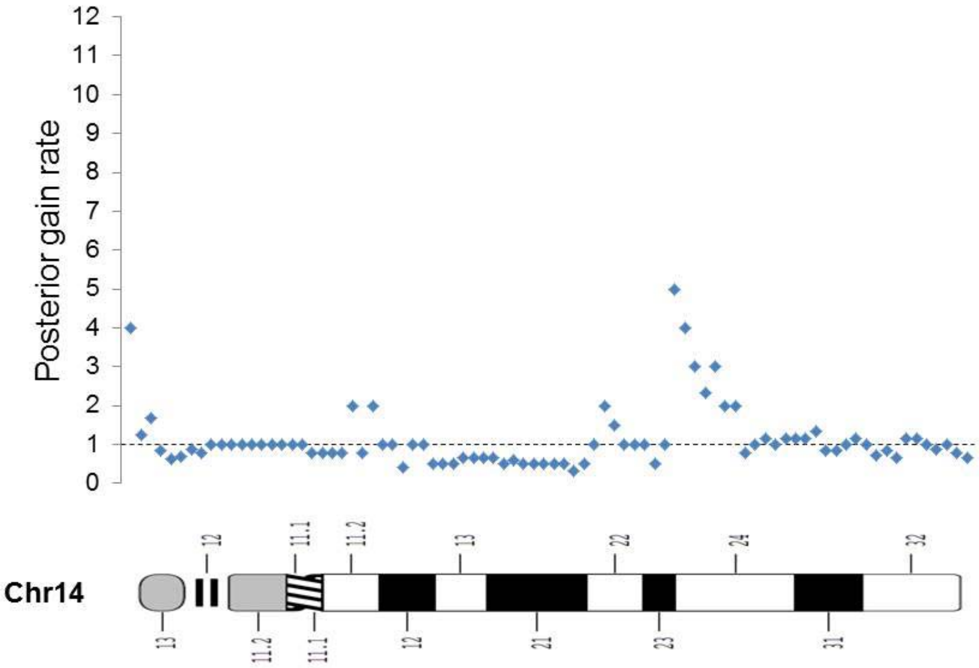

Fig N (related to Fig 2). Posterior gain rate (M:F) plotted serially on chromosome 15 using available genes from DECIPHER. Dashed line depicts equal gain rate.

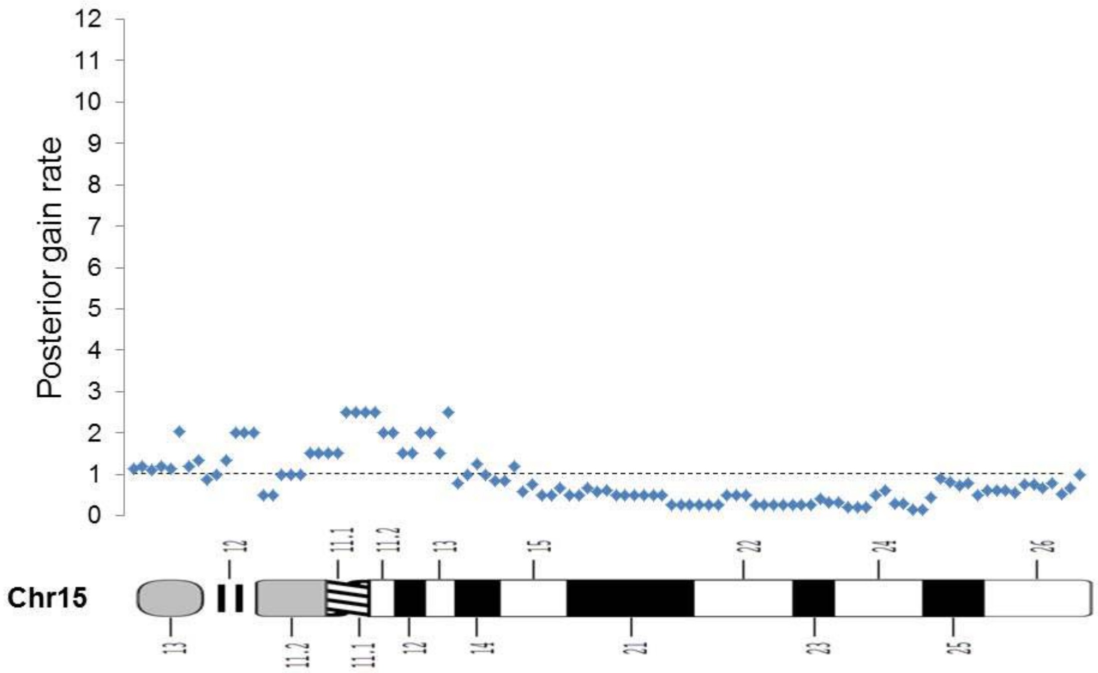

Fig O (related to Fig 2). Posterior gain rate (M:F) plotted serially on chromosome 16 using available genes from DECIPHER. Dashed line depicts equal gain rate.

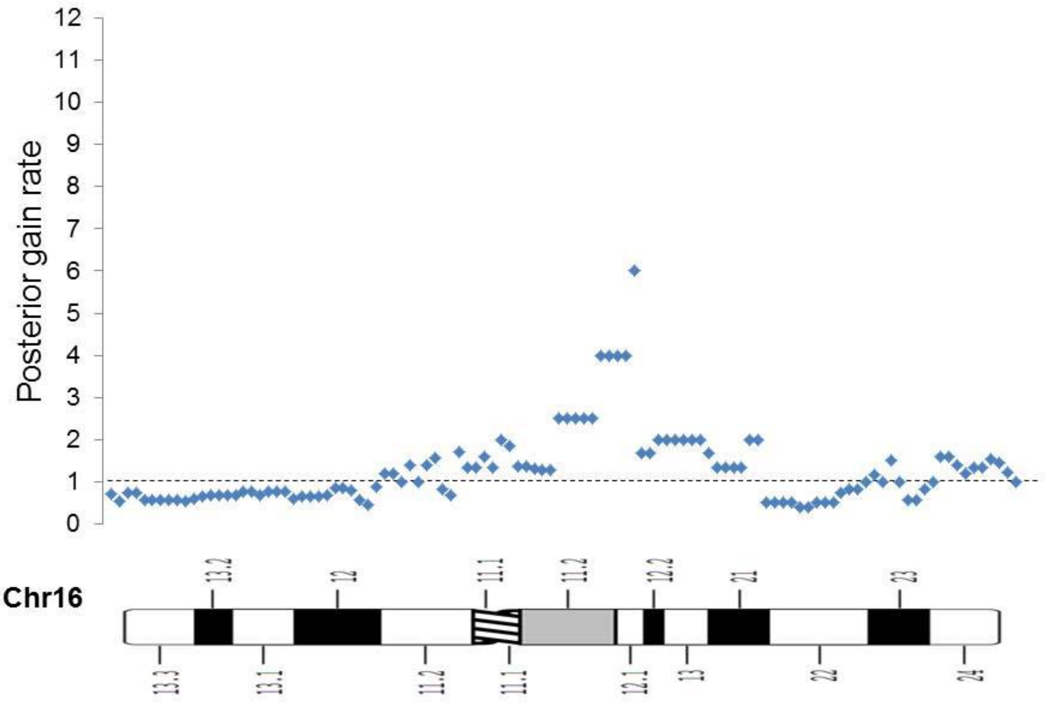

Fig P (related to Fig 2). Posterior gain rate (M:F) plotted serially on chromosome 17 using available genes from DECIPHER. Dashed line depicts equal gain rate.

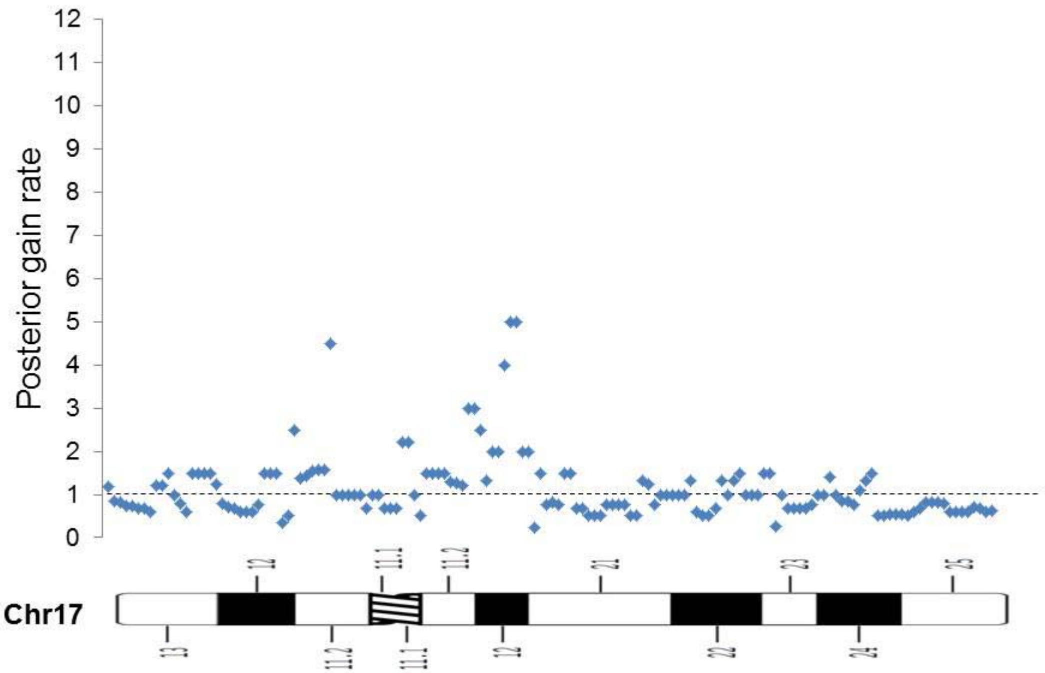

Fig Q (related to Fig 2). Posterior gain rate (M:F) plotted serially on chromosome 18 using available genes from DECIPHER. Dashed line depicts equal gain rate.

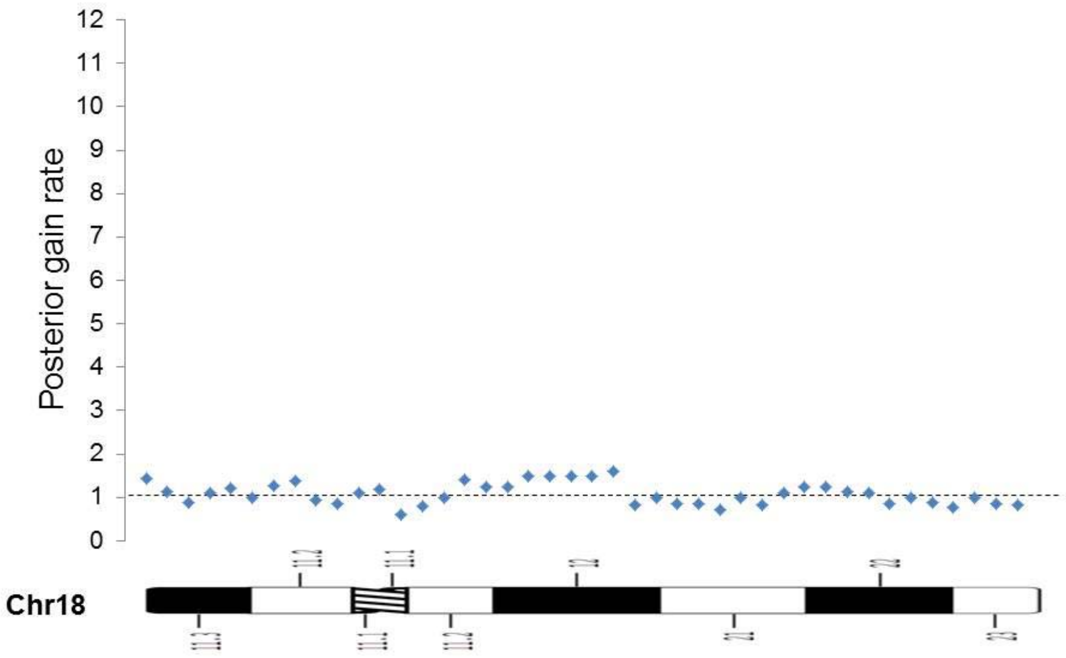

Fig R (related to Fig 2). Posterior gain rate (M:F) plotted serially on chromosome 20 using available genes from DECIPHER. Dashed line depicts equal gain rate.

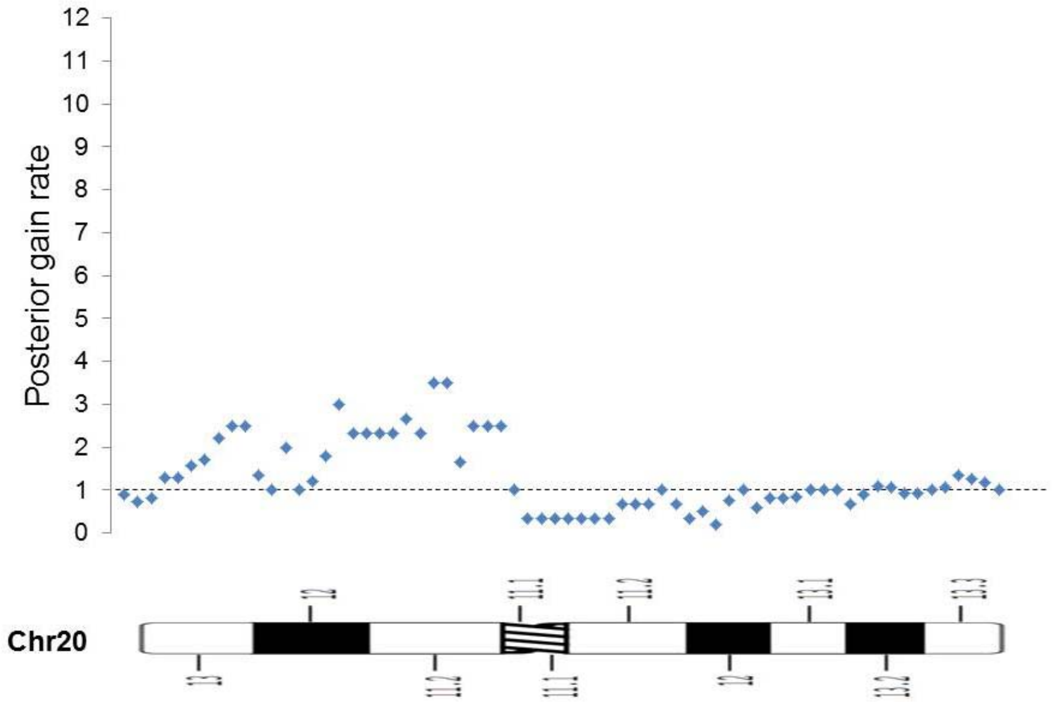

Fig S (related to Fig 2). Posterior gain rate (M:F) plotted serially on chromosome 21 using available genes from DECIPHER. Dashed line depicts equal gain rate.

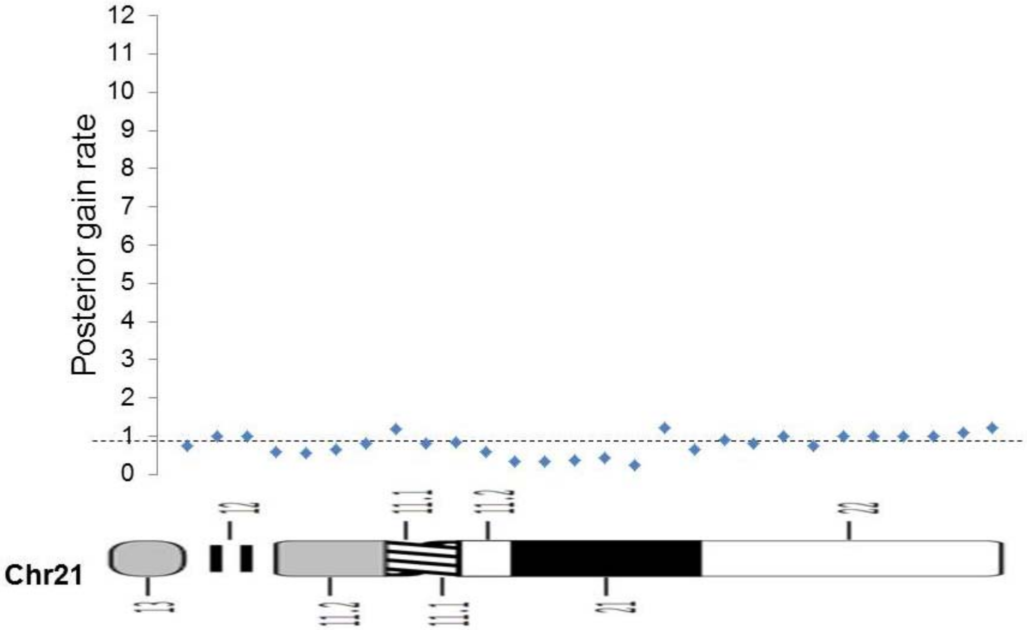

Fig T (related to Fig 2). Posterior gain rate (M:F) plotted serially on chromosome 22 using available genes from DECIPHER. Dashed line depicts equal gain rate.

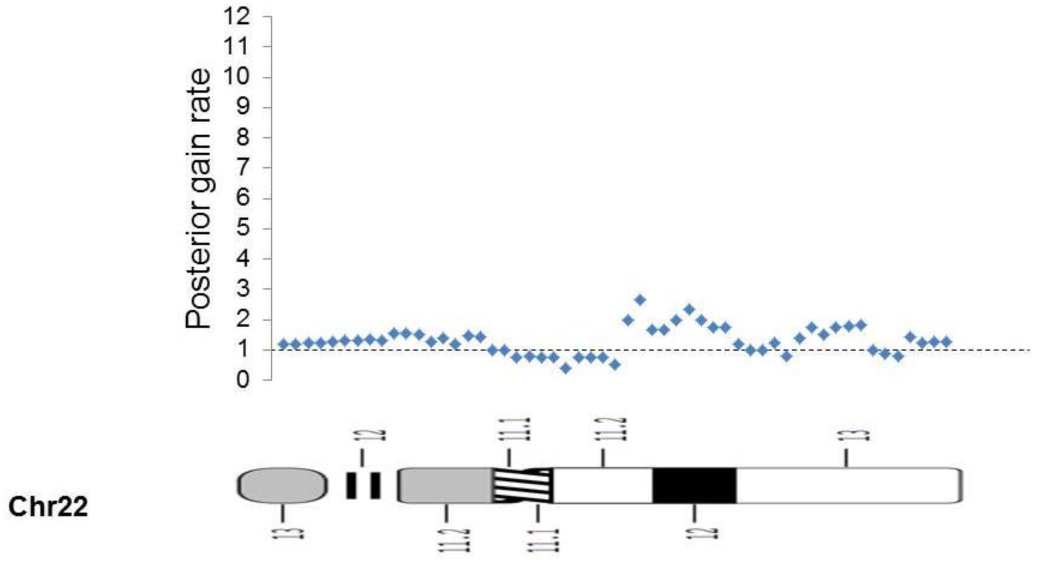

Supplement: S1 Fig — Posterior gain rate (M:F) plotted serially on chromosomes 2–18 and 20–22 (Figs A-T) using available genes from DECIPHER. Dashed line depicts equal gain rate. (PDF) [file pone.0170403.s001.pdf]
