# Supplementary material for: Embryonic loss of human females with partial trisomy 19 identifies region critical for the single active X
Source: PLoS One. 2017 Apr 12;12(4):e0170403. doi: 10.1371/journal.pone.0170403 (PMC5389809; doi:10.1371/journal.pone.0170403)
Supplement: S2 Fig — Permutation testing reveals that skewing in the 19p region is highly significant (p<10−11). Calculations show that even after 107 random simulations, it would require 104−105 more simulations to observe such a skewing at least once. (DOCX) [file pone.0170403.s002.docx]

**S2 Fig.** (related to Fig 3) Permutation testing reveals that skewing in the 19p region is highly significant (p<10^-11^). Calculations show that even after 10^7^ random simulations, it would require 10^4^-10^5^ more simulations to observe such a skewing at least once.
