# Supplementary material for: Embryonic loss of human females with partial trisomy 19 identifies region critical for the single active X
Source: PLoS One. 2017 Apr 12;12(4):e0170403. doi: 10.1371/journal.pone.0170403 (PMC5389809; doi:10.1371/journal.pone.0170403)
Supplement: S1 Table — Details of data in Fig 3, including sex ratio of total variants, duplications and deletions and number of genes assayed on Chromosome 19 in 500 KB bins. (DOCX) [file pone.0170403.s003.docx]

**S1 Table** (related to Fig 3). Details of data in Fig 3, including sex ratio of total variants, duplications and deletions,

and number of genes assayed on Chromosome 19 in 500 KB bins.

| **Chromosome Band** | **Regional Bin** | | **Total Variants** | | **Duplications** | | **Deletions** | | **Number of Genes** |
| --- | --- | --- | --- | --- | --- | --- | --- | --- | --- |
|  | **Start (KB)** | **Finish (KB)** | **F** | **M** | **F** | **M** | **F** | **M** |  |
| 19p13.3 | 100,000 | 500,000 | **11** | **15** | **2** | **7** | **6** | **5** | 15 |
|  | 500,000 | 1,000,000 | **15** | **18** | **4** | **10** | **6** | **4** | 27 |
|  | 1,000,000 | 1,500,000 | **15** | **21** | **2** | **11** | **6** | **4** | 28 |
|  | 1,500,000 | 2,000,000 | **5** | **20** | **1** | **13** | **1** | **3** | 19 |
|  | 2,000,000 | 2,500,000 | **16** | **15** | **4** | **13** | **4** | **0** | 22 |
|  | 2,500,000 | 3,000,000 | **10** | **15** | **4** | **11** | **4** | **2** | 14 |
|  | 3,000,000 | 3,500,000 | **15** | **18** | **5** | **9** | **9** | **5** | 10 |
|  | 3,500,000 | 4,000,000 | **17** | **26** | **3** | **10** | **13** | **10** | 25 |
| 19p13.3 | 4,000,000 | 4,500,000 | **19** | **26** | **2** | **10** | **13** | **11** | 19 |
| 19 p13.3 | 4,500,000 | 5,000,000 | **20** | **22** | **1** | **13** | **9** | **7** | 17 (UHRF1) |
| 19p13.3 | 5,000,000 | 5,500,000 | **2** | **17** | **1** | **11** | **2** | **3** | 5 (KDM4B, PTPRS) |
| 19p13.3 | 5,500,000 | 6,000,000 | **1** | **14** | **1** | **10** | **0** | **2** | 20 |
| 19p13.3 | 6,000,000 | 6,500,000 | **1** | **8** | **1** | **8** | **0** | **0** | 15 |
| 19p13.3 &13.2 | 6,500,000 | 7,000,000 | **4** | **11** | **1** | **10** | **0** | **0** | 12 |
| 19p13.2 | 7,000,000 | 7,500,000 | **6** | **10** | **1** | **10** | **1** | **0** | 7 |
| 19p13.2 | 7,500,000 | 8,000,000 | **3** | **8** | **1** | **5** | **1** | **0** | 30 |
|  | 8,000,000 | 8,500,000 | **2** | **5** | **1** | **4** | **1** | **1** | 14 |
| 19p13.2 | 8,500,000 | 9,000,000 | **4** | **5** | **1** | **4** | **0** | **0** | 12 |
| 19p13.2 | 9,000,000 | 9,500,000 | **1** | **5** | **1** | **4** | **0** | **0** | 21 |
| 19p13.2 | 9,500,000 | 10,000,000 | **1** | **7** | **1** | **5** | **0** | **0** | 16 (7ZNFFBxl12, UBL5) |
| 19p13.2 | 10,000,000 | 10,500,000 | **3** | **6** | **0** | **4** | **3** | **0** | 11 (DNMT1) |
| 19p13.2 | 10,500,000 | 11,000,000 | **6** | **9** | **0** | **6** | **5** | **1** | 22 |
| 19p13.2 | 11,000,000 | 11,500,000 | **14** | **13** | **1** | **5** | 4 | **0** | 18 (166kb dup mat inherited) |
| Midpoint 19p13.2 | 11,500,000 | 12,000,000 | **4** | **11** | **0** | **7** | 3 | **3** | 20 (3 ZNF ELAV 3 SWSAP1) |
| 19p13.2 | 12,000,000 | 12,500,000 | **4** | **13** | **0** | **7** | 2 | **3** | 19 (13 ZNF cluster) |
| 19p13.2 | 12,500,000 | 13,000,000 | **8** | **11** | **4** | **10** | **1** | **4** | 27  6 (ZNF) |
| 19p13.2 | 13,500,000 | 14,000,000 | **14** | **10** | **3** | **5** | **7** | **3** | 13 |
| 19p13.2 | 14,000,000 | 14,500,000 | **11** | **9** | **2** | **6** | **6** | **2** | 16 |
| 19p13.12 | 14,500,000 | 15,000,000 | **22** | **10** | **3** | **6** | **6** | **3** | 22 (ZNF 333) |
|  | 15,000,000 | 15,500,000 | **9** | **10** | **2** | **4** | **5** | **5** | 17 |
|  | 15,500,000 | 16,000,000 | **9** | **6** | **2** | **3** | **5** | **2** | 20 |
| 19p13.11 | 16,000,000 | 16,500,000 | **6** | **5** | **1** | **2** | **2** | **2** | 16 |
|  | 16,500,000 | 17,000,000 | **3** | **6** | **1** | **1** | **0** | **2** | 13 |
|  | 17,000,000 | 17,500,000 | **3** | **5** | **1** | **2** | **0** | **1** | 18 |
|  | 17,500,000 | 18,000,000 | **5** | **5** | **1** | **2** | **0** | **1** | 17 |
|  | 18,000,000 | 18,500,000 | **3** | **4** | **1** | **2** | **1** | **2** | 22 |
|  | 18,500,000 | 19,000,000 | **19** | **5** | **2** | **2** | **1** | **2** | 17 (many females with 142kb gain inherited from normal parent) |
|  | 19,000,000 | 19,500,000 | **5** | **3** | **2** | **1** | **2** | **2** | 22 |
|  | 19,500,000 | 20,000,000 | **1** | **4** | **1** | **2** | **0** | **1** | 16 |
|  | 20,000,000 | 20,500,000 | **2** | **6** | **1** | **2** | **0** | **2** | 6 (5 ZNF) |
|  | 20,500,000 | 21,000,000 | **3** | **7** | **1** | **5** | **1** | **1** | 8 (2 ZNF) |
|  | 21,000,000 | 21,500,000 | **3** | **6** | **1** | **4** | **1** | **1** | 13 (5 ZNF) |
|  | 21,500,000 | 22,000,000 | **1** | **5** | **1** | **3** | **0** | **1** | 8 (3 ZNF) |
| 19p12 | 22,500,000 | 23,000,000 | **1** | **4** | **1** | **2** | **0** | **1** | 10 (3 ZNF) |
|  | 23,000,000 | 23,500,000 | **1** | **4** | **1** | **2** | **0** | **1** | 9 (3 ZNF) |
|  | 23,500,000 | 24,000,000 | **3** | **11** | **2** | **3** | **0** | **5** | 8 (3 ZNF) |
|  | 24,000,000 | 24,500,000 | **3** | 11 | **2** | 3 | **1** | **5** | 6 (2 ZNF) |
| 19p11 | 24,500,000 | 25,000,000 | **2** | 3 | **1** | 2 | **0** | **0** | 0 |
|  | 25,000,000 | 25,500,000 | **1** | 2 | **0** | 1 | **0** | **0** | 0 |
|  | 25,500,000 | 26,000,000 | **1** | 2 | **0** | 2 | **0** | **0** | 0 |
|  | 26,000,000 | 26,500,000 | **1** | 2 | **0** | 2 | **0** | **0** | 0 |
| CENTROMERE | 26,500,000 | 27,000,000 | **1** | 2 | **0** | 2 | **0** | **0** | 0 |
| 19q11 | 27,000,000 | 27,500,000 | **1** | 2 | **0** | 2 | **0** | **0** | 0 |
|  | 27,500,000 | 28,000,000 | **2** | 2 | **0** | 2 | **0** | **0** | 0 |
|  | 28,000,000 | 28,500,000 | **6** | 7 | **1** | 4 | **0** | **1** | 2 |
|  | 28,500,000 | 29,000,000 | **5** | 7 | **1** | 4 | **0** | **1** | 0 |
| 19q12 | 29,000,000 | 29,500,000 | **2** | 5 | **1** | 3 | **0** | **1** | 2 |
|  | 29,500,000 | 30,000,000 | **3** | 6 | **1** | 4 | **1** | **1** | 2 |
|  | 30,000,000 | 30,500,000 | **6** | 7 | **3** | 5 | **1** | **1** | 7 |
|  | 30,500,000 | 31,000,000 | **20** | 13 | **3** | 6 | **1** | **2** | 3 |
|  | 31,000,000 | 31,500,000 | **21** | 12 | **3** | 5 | **2** | **4** | 1 |
| 19q12 | 31,500,000 | 32,000,000 | **7** | 12 | **3** | 4 | **2** | **7** | 1 |
| 19q12-q13.11 | 32,000,000 | 32,500,000 | **5** | 13 | **2** | 5 | **1** | **6** | 2 |
|  | 32,500,000 | 33,000,000 | **5** | 13 | **2** | 5 | **1** | **6** | 3 |
| 19q13.11 | 33,000,000 | 33,500,000 | **4** | 13 | **2** | 4 | **2** | **7** | 13 |
|  | 33,500,000 | 34,000,000 | **4** | 12 | **2** | 5 | **2** | **5** | 11 |
|  | 34,000,000 | 34,500,000 | **4** | 9 | **2** | 4 | **2** | **4** | 5 |
|  | 34,500,000 | 35,000,000 | **9** | 10 | **2** | 5 | **5** | **3** | 12 |
|  | 35,000,000 | 35,500,000 | **8** | 8 | **1** | 4 | **5** | **2** | 12 |
|  | 35,500.000 | 36,000,000 | **6** | **8** | **1** | **3** | **3** | **3** | 25 |
|  | 36,000,000 | 36,500,000 | **6** | **9** | **1** | **3** | **2** | **2** | 36 |
|  | 36,500,000 | 37,000,000 | **6** | **7** | **1** | **3** | **3** | **1** | 17 |
|  | 37,000,000 | 37,500,000 | **5** | **8** | **1** | **3** | **2** | **3** | 12 (10 ZNF) |
|  | 37,500,000 | 38,000,000 | **5** | **6** | **1** | **2** | **1** | **1** | 9 (7 ZNF) |
| 19q13.12- 13.2 | 38,000,000 | 38,500,000 | **4** | **4** | **1** | **2** | **1** | **0** | 19 (WDR 87, 7 ZNF) |
|  | 38,500,000 | 39,000,000 | **3** | **4** | **1** | **2** | **1** | **0** | 15 |
|  | 39,000,000 | 39,500,000 | **3** | **3** | **1** | **2** | **0** | **0** | 19 (SIRT2, HNRNPL) |
|  | 39,500,000 | 40,000,000 | **3** | **4** | **1** | **2** | **0** | **0** | 24 (FBOXP, ZNFRP) |
|  | 40,000,000 | 40,500,000 | **1** | **4** | **1** | **3** | **0** | **0** | 16 (DYRK1B, LEUTX) |
|  | 40,500,000 | 41,000,000 | **1** | **6** | **1** | **3** | **0** | **0** | 18 |
|  | 41,000,000 | 41,500,000 | **0** | **9** | **0** | **5** | **0** | **0** | 22 (SHKBP1,SNRPA, RAB4B, C19ORF54) |
|  | 41,500,000 | 42,000,000 | **0** | **4** | **0** | **1** | 0 | **0** | 21 (TGFB1,HNRPUL1,CCDC97) |
|  | 42,000,000 | 42,500,000 | **7** | **5** | **0** | **1** | 1 | **0** | 18 |
|  | 42,500,000 | 43,000,000 | **5** | **3** | **1** | **2** | 2 | **1** | 19 (2Z NF, OCT2) |
|  | 43,000,000 | 43,500,000 | **3** | **4** | **1** | **3** | 1 | **1** | 15 |
|  | 43,500,000 | 44,000,000 | **4** | **1** | **1** | **1** | 1 | **0** | 15 |
|  | 44,000,000 | 44,500,000 | **2** | **1** | **1** | **1** | 0 | **0** | 21 (8 ZNF) |
|  | 44,500,000 | 45,000,000 | **1** | **1** | **0** | **1** | **0** | **0** | 20 (15 ZNF) |
|  | 45,000,000 | 45,500,000 | **1** | **6** | **0** | **1** | **0** | **0** | 19 |
|  | 45,500,000 | 46,000,000 | **12** | **10** | **3** | **3** | **0** | **0** | 22 |
| 19q13.32 | 46,000,000 | 46,500,000 | **3** | **6** | **1** | **1** | **0** | **2** | 25 |
|  | 46,500,000 | 47,000,000 | **4** | **5** | **1** | **3** | **0** | **2** | 18 |
|  | 47,000,000 | 47,500,000 | **6** | **7** | **2** | **2** | **1** | **2** | 16 |
|  | 47,500,000 | 48,000,000 | **10** | **8** | **6** | **3** | **1** | **2** | 18 |
|  | 48,000,000 | 48,500,000 | **22** | **11** | **8** | **3** | **1** | **2** | 16 |
|  | 48,500,000 | 49, 000,000 | **19** | **10** | **7** | **4** | **0** | **0** | 17 (ZNF114) |
|  | 49,000,000 | 49,500,000 | **5** | **4** | **5** | **3** | **0** | **0** | 30 |
|  | 49,500,000 | 50,000,000 | **10** | **2** | **8** | **2** | **0** | **0** | 40 |
|  | 50,000,000 | 50,500,000 | **10** | **3** | **5** | **2** | **0** | **1** | 35 |
|  | 50,500,000 | 51,000,000 | **12** | **8** | **5** | **5** | **0** | **2** | 14 |
|  | 51,000,000 | 51,500,000 | **6** | **8** | **5** | **4** | **0** | **3** | 24 |
|  | 51,500,000 | 52,000,000 | **6** | **6** | **5** | **2** | **0** | **3** | 30 |
|  | 52,000,000 | 52,500,000 | **7** | **13** | **5** | **8** | **0** | **3** | 25 (6 ZNF) |
| q13.41 | 52,500,000 | 53,000,000 | **12** | **15** | **6** | **9** | **2** | **1** | 17 (13 ZNF) |
|  | 53,000,000 | 53,500,000 | **8** | **10** | **7** | **6** | **1** | **1** | 16 (10 ZNF) |
|  | 53,500,000 | 54,000,000 | **7** | **12** | **5** | **7** | **2** | **2** | 24 (10 ZNF) |
|  | 54,000,000 | 54,500,000 | **9** | **12** | **8** | **11** | **1** | **0** | 70 |
|  | 54,500,000 | 55,000,000 | **14** | **18** | **11** | **10** | **0** | **0** | 29 |
|  | 55,000,000 | 55,500,000 | **14** | **17** | **11** | **10** | **0** | **0** | 29 |
| 19q13.42 | 55,500,000 | 56,000,000 | **7** | **11** | **6** | **6** | **1** | **2** | 26 |
| 19q42- q13.43 | 56,000,000 | 56,500,000 | **12** | **15** | **8** | **10** | **1** | **0** | 22 (7 ZNF) |
| 19q13.43 | 56,500,000 | 57,000,000 | **11** | **18** | **9** | **13** | **1** | **0** | 16 (7 ZNF) |
| 19q13.43 | 57,000,000 | 57,500,000 | **9** | **13** | **8** | **10** | **1** | **0** | 12 (6 ZNF) |
|  | 57,500,000 | 58,000,000 | **7** | **11** | **7** | **7** | **0** | **1** | 19 (10 ZNF) |
|  | 58,000,000 | 58,500,000 | **7** | **10** | **6** | **4** | **1** | **0** | 24 (21 ZNF) |
|  | 58,500,000 | 59,000,000 | **6** | **8** | **5** | **4** | **0** | **0** | 29 (15 ZNF) |
